# Supplementary figures and images for: Inhibition of RPS6K reveals context-dependent Akt activity in luminal breast cancer cells
Source: PLoS Comput Biol. 2021 Jun 30;17(6):e1009125. doi: 10.1371/journal.pcbi.1009125 (PMC8277016; doi:10.1371/journal.pcbi.1009125)

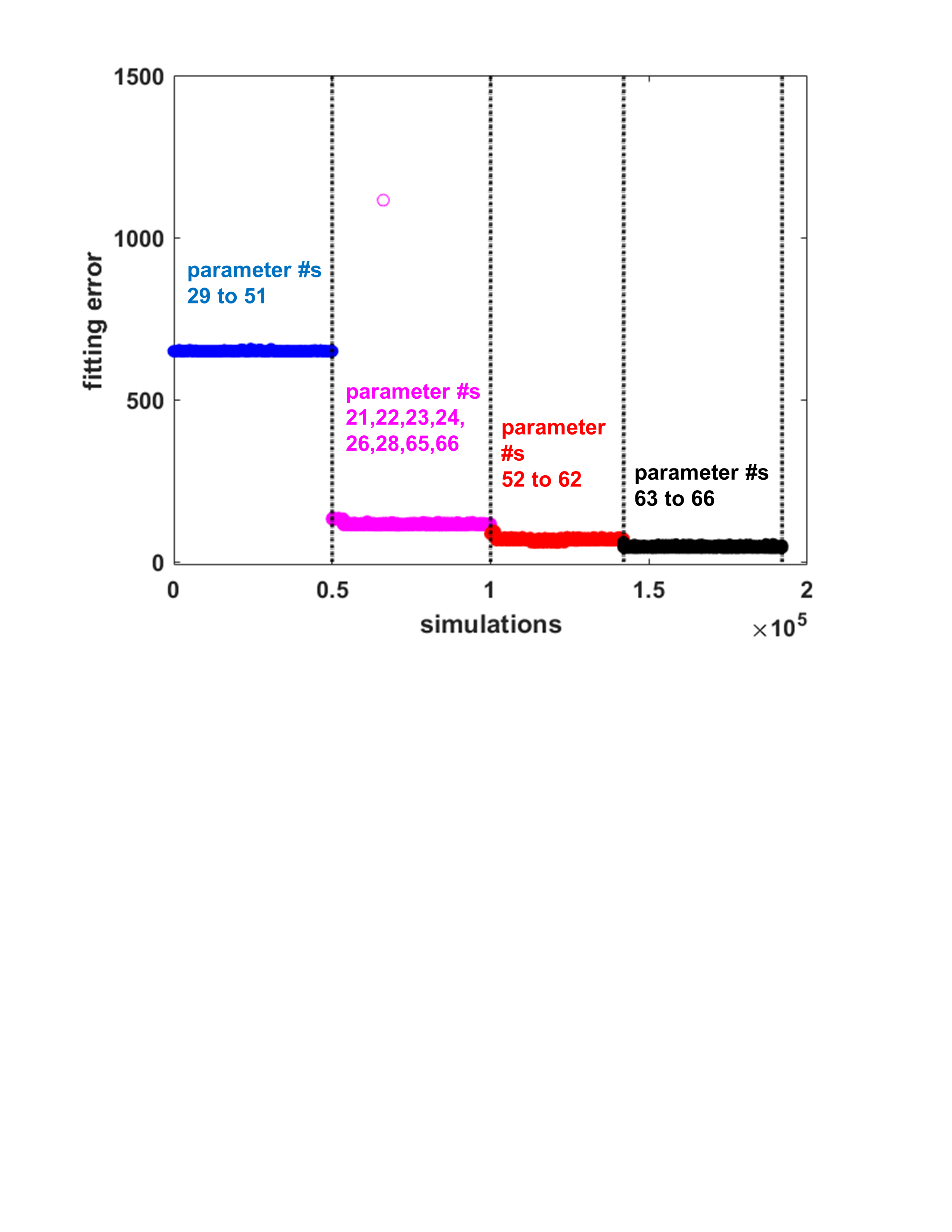

Supplement: S1 Fig — The trend shows a decrease of fitting error over the selected parameters. However, there are cases of acceptance for slightly worse performing parameter values and a very poor performing parameter value set is chosen once. Each dot above is a simulation error of a single parameter set, where only the values of the mentioned list of parameters are varied from the starting condition. (TIF) [file pcbi.1009125.s001.tif]

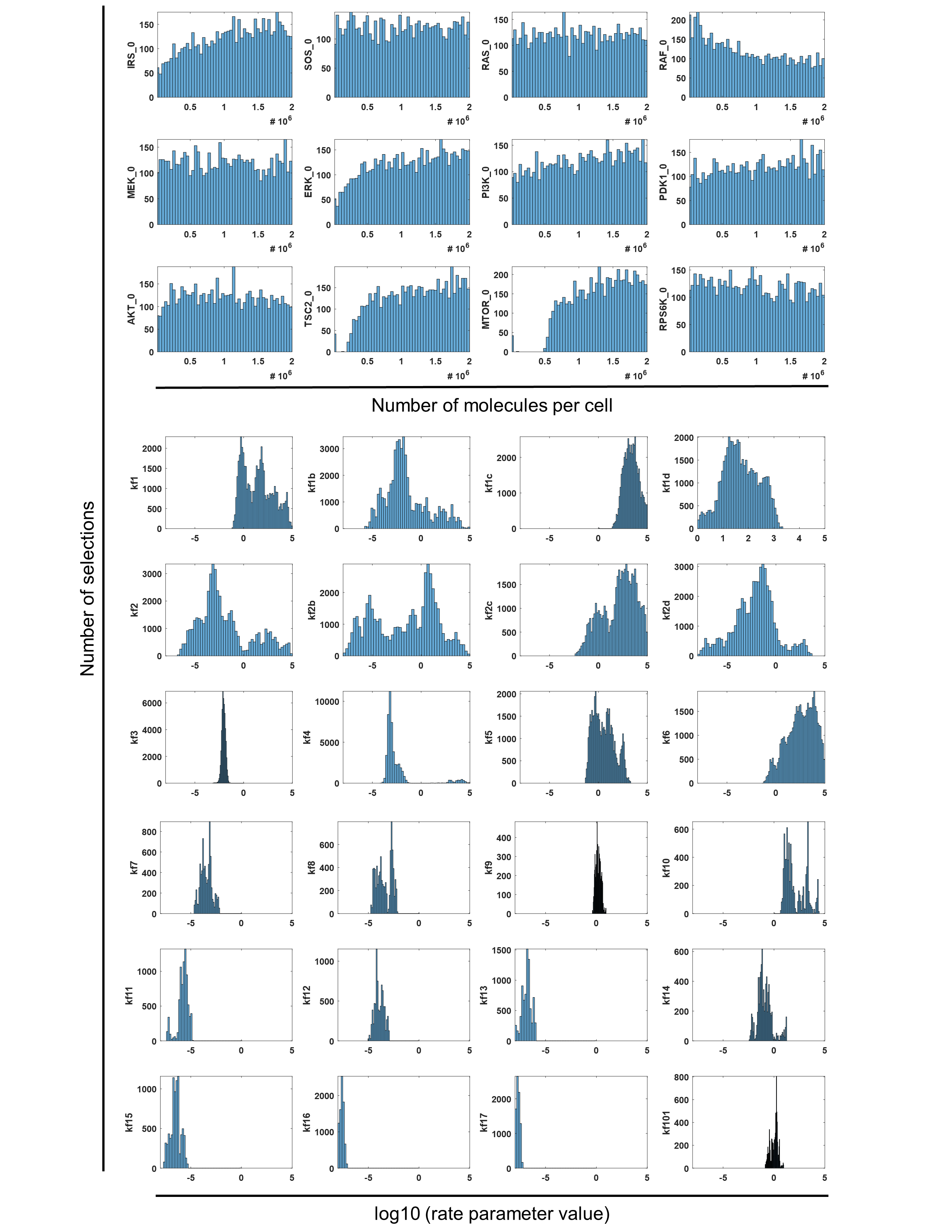

Supplement: S2 Fig — Each subpanel histogram depicts posterior parameter distribution for one parameter in the model. Top 12 panels are an example on total protein levels, showing distributions from ~6000 parameter sets. The histograms for rate parameter values (from ~50000 parameter sets) are shown in log10 values in x-axes. Continued on S3 Fig. (TIF) [file pcbi.1009125.s002.tif]

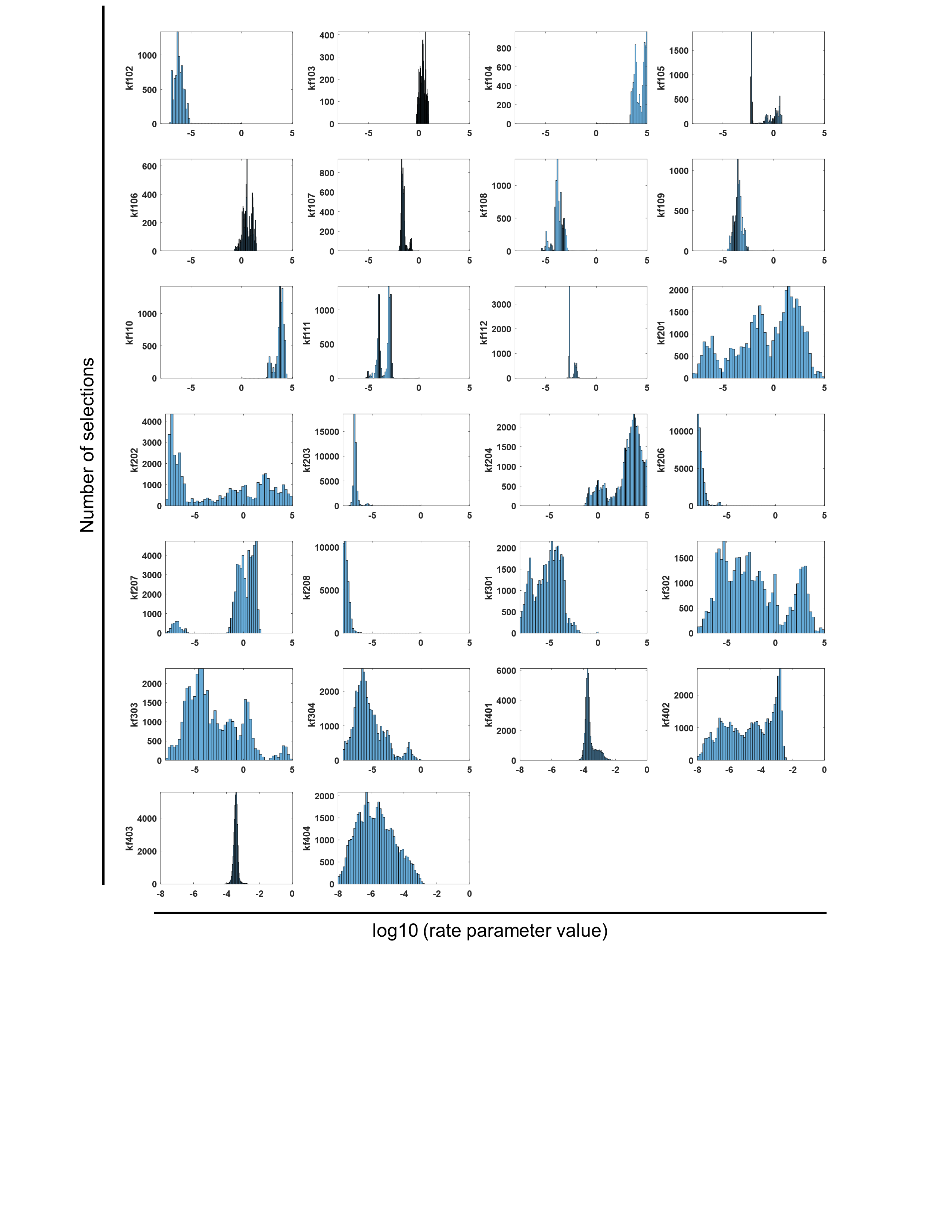

Supplement: S3 Fig — Each subpanel histogram depicts posterior parameter distribution for one parameter in the model. The histogram values (from ~50000 parameter sets) are shown in log10 values in x-axes. (TIF) [file pcbi.1009125.s003.tif]

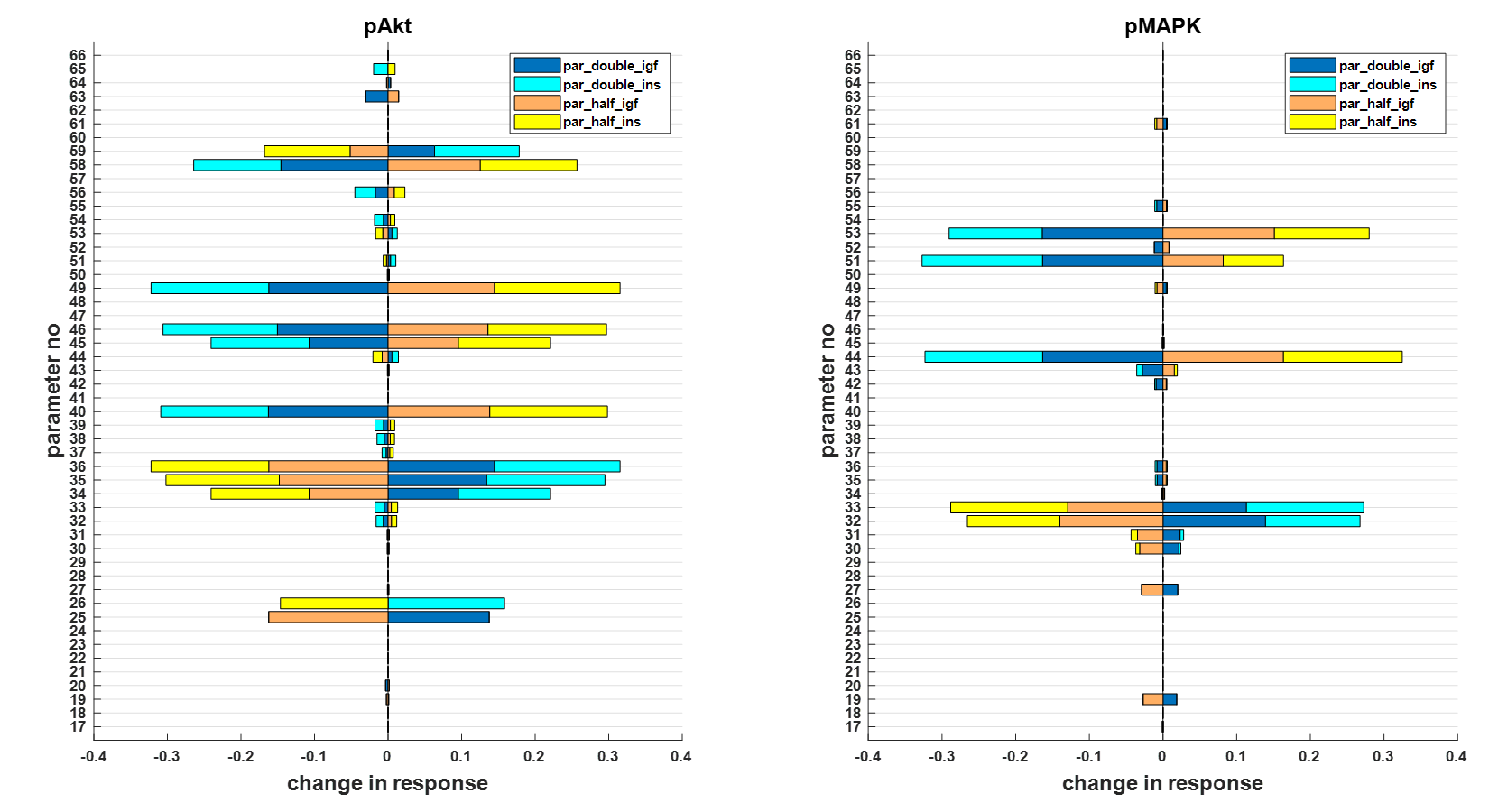

Supplement: S4 Fig — Changing the individual parameter values up or down 2X causes changes in Akt and MAPK phosphorylation levels. The values reported indicate the ratio of perturbed model response protein level to the level of the protein of un-perturbed model at 5min. Dark and light blue colors represent doubled parameter rate conditions with IGF1 and insulin stimulations, respectively. Orange and yellow colors represent halved parameter rate conditions with IGF1 and insulin stimulations, respectively. Bars are shown as concatenated for IGF1 and insulin conditions, where applicable. The two opposite parameter manipulations result in opposite changes in the phosphorylation levels, with very similar changes imposed in IGF1 and insulin. (TIF) [file pcbi.1009125.s004.tif]

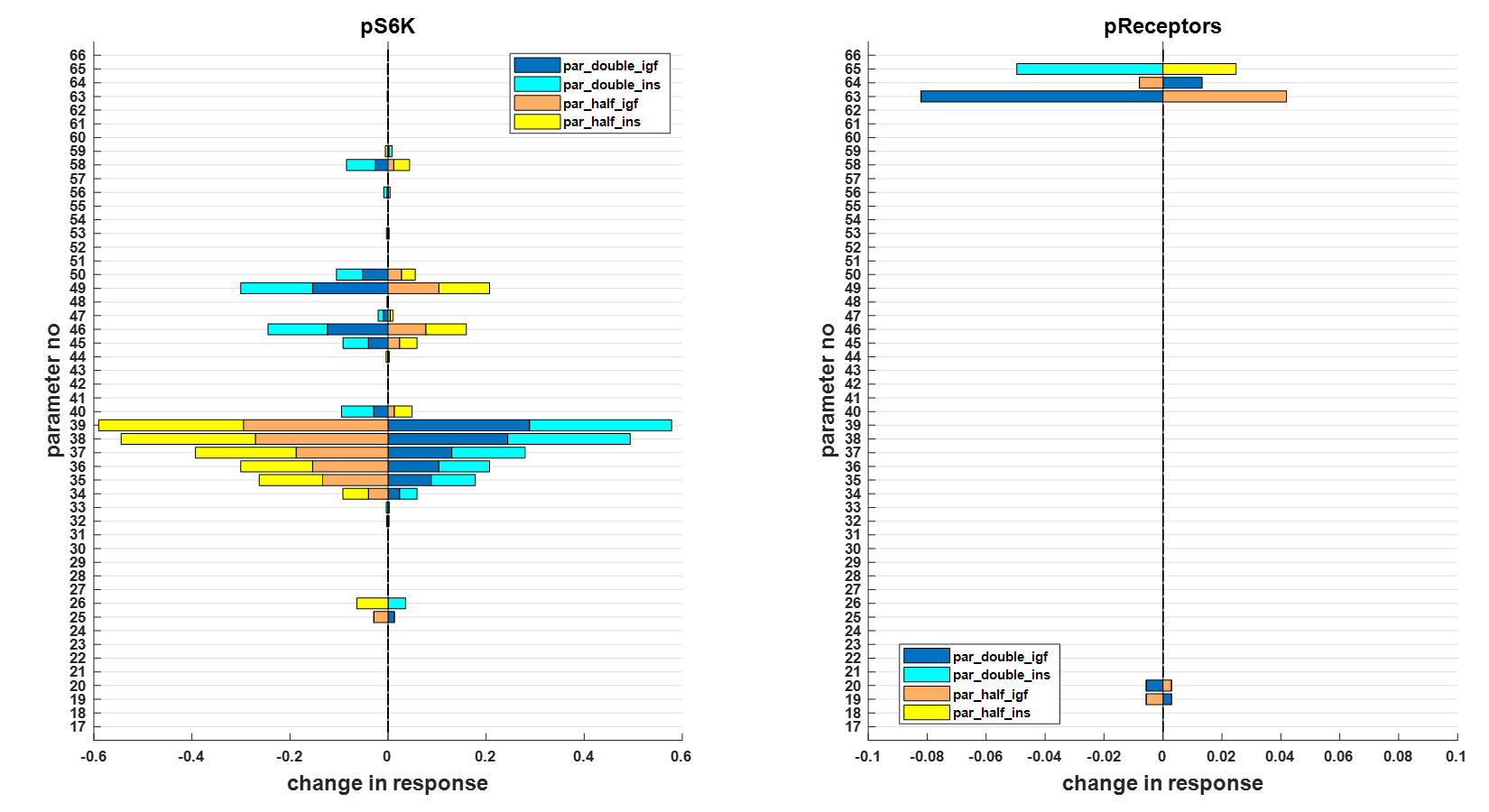

Supplement: S5 Fig — Changing the individual parameter values up or down 2X causes changes in S6 kinase and receptor phosphorylation levels. The values reported indicate the ratio of perturbed model response protein level to the level of the protein of un-perturbed model at 5min. Dark and light blue colors represent doubled parameter rate conditions with IGF1 and insulin stimulations, respectively. Orange and yellow colors represent halved parameter rate conditions with IGF1 and insulin stimulations, respectively. Bars are shown as concatenated for IGF1 and insulin conditions, where applicable. The two opposite parameter manipulations result in opposite changes in the phosphorylation levels, with very similar changes imposed in IGF1 and insulin. (TIF) [file pcbi.1009125.s005.tif]

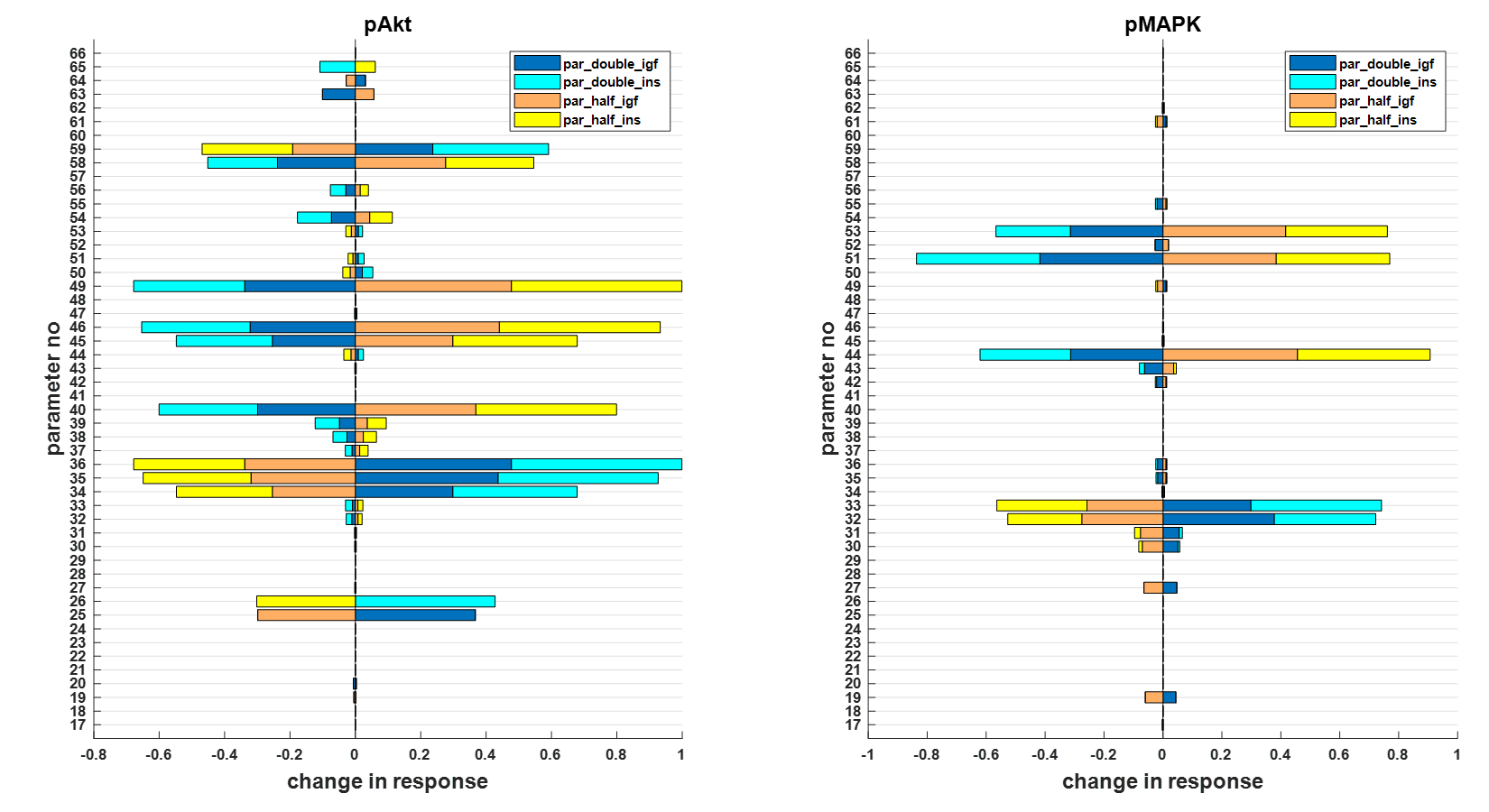

Supplement: S6 Fig — Changing the individual parameter values up or down 2X causes changes in Akt and MAPK phosphorylation levels. The values reported indicate the ratio of the AUC of the corresponding protein in perturbed model to the AUC of that protein in un-perturbed model, from time point zero to 30 min of stimulation. Dark and light blue colors represent doubled parameter rate conditions with IGF1 and insulin stimulations, respectively. Orange and yellow colors represent halved parameter rate conditions with IGF1 and insulin stimulations, respectively. Bars are shown as concatenated for IGF1 and insulin conditions, where applicable. The two opposite parameter manipulations result in opposite changes in the phosphorylation levels, with very similar changes imposed in IGF1 and insulin. (TIF) [file pcbi.1009125.s006.tif]

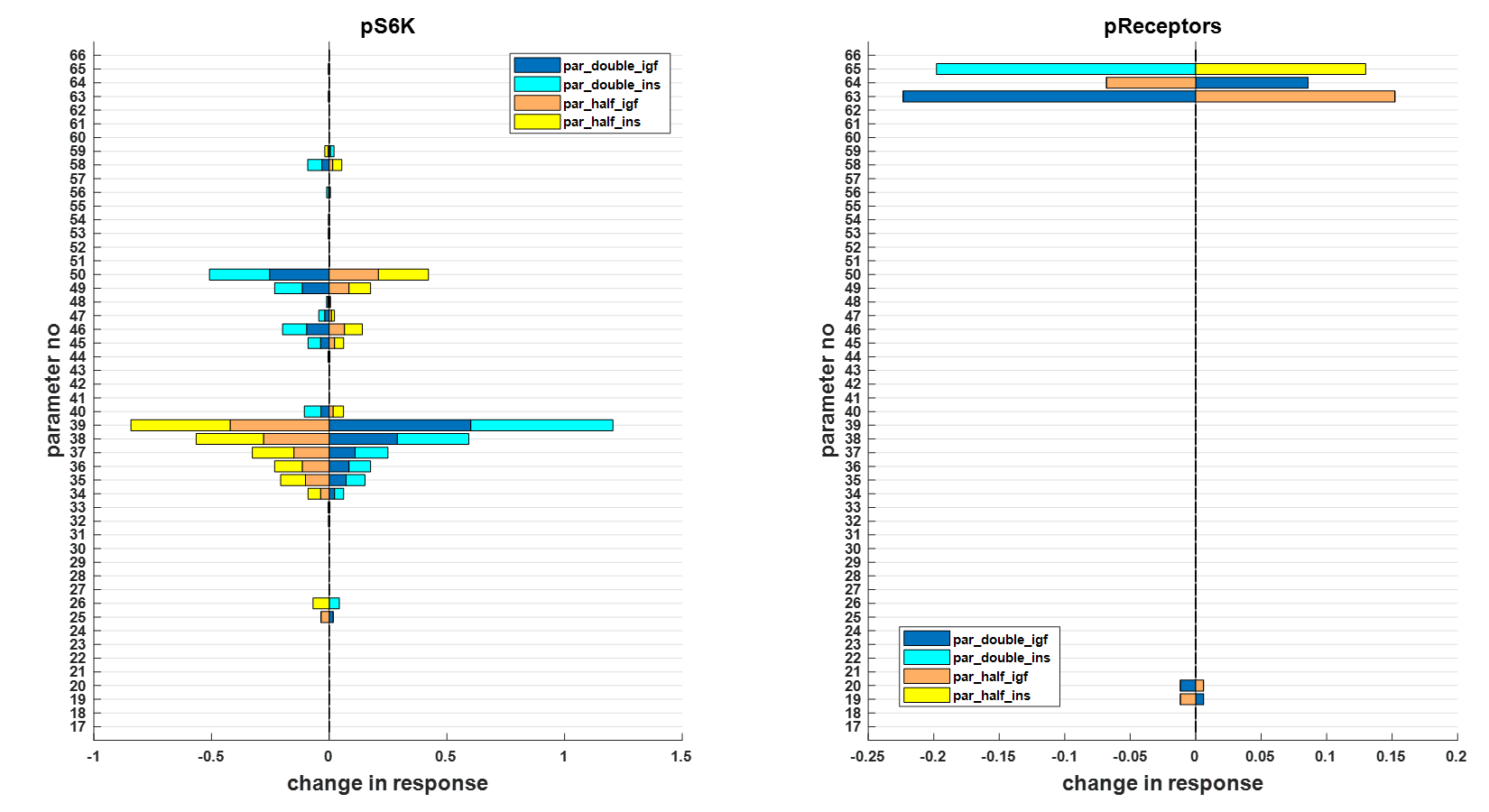

Supplement: S7 Fig — Changing the individual parameter values up or down 2X causes changes S6 kinase and receptor phosphorylation levels. The values reported indicate the ratio of the AUC of the corresponding protein in perturbed model to the AUC of that protein in un-perturbed model, from time point zero to 30 min of stimulation. Dark and light blue colors represent doubled parameter rate conditions with IGF1 and insulin stimulations, respectively. Orange and yellow colors represent halved parameter rate conditions with IGF1 and insulin stimulations, respectively. Bars are shown as concatenated for IGF1 and insulin conditions, where applicable. The two opposite parameter manipulations result in opposite changes in the phosphorylation levels, with very similar changes imposed in IGF1 and insulin. (TIF) [file pcbi.1009125.s007.tif]

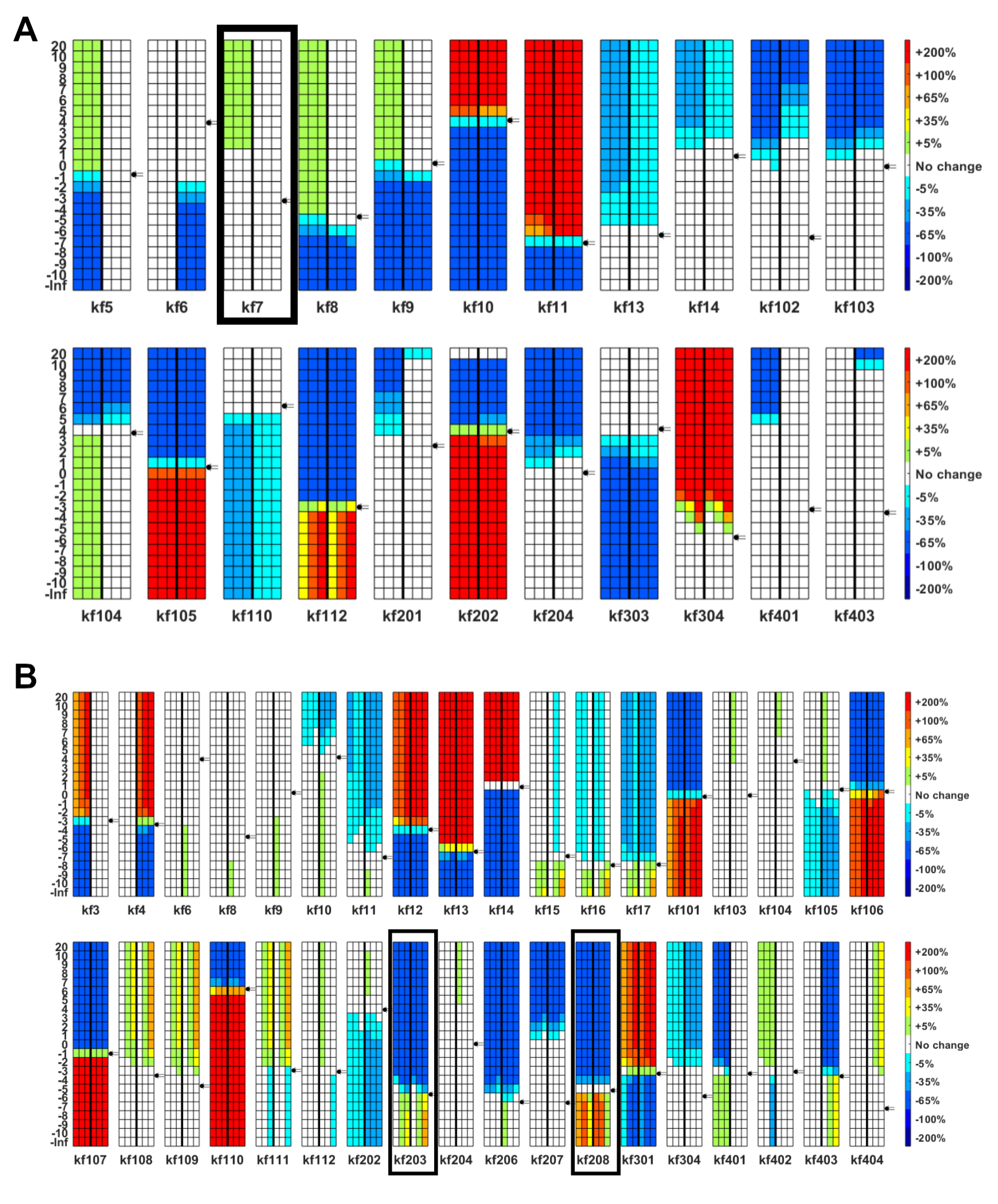

Supplement: S8 Fig — The ODE model output of pMAPK (A) and pAkt (B) levels are determined by setting and changing the value of each parameter individually, from zero to infinity. Each box above is for a parameter, and each column is for one time point response of 5, 10, and 30min. Left three columns are from IGF1 stimulation results and the right three columns are from insulin stimulated simulations. Rows represent the log10 of set parameter value. The colors represent the log2 fold change from un-perturbed model output. Red and blue respectively indicate up and down regulation of the specified phosphorylation. The dotted arrow heads indicate the unperturbed model parameter values in log2. (TIF) [file pcbi.1009125.s008.tif]

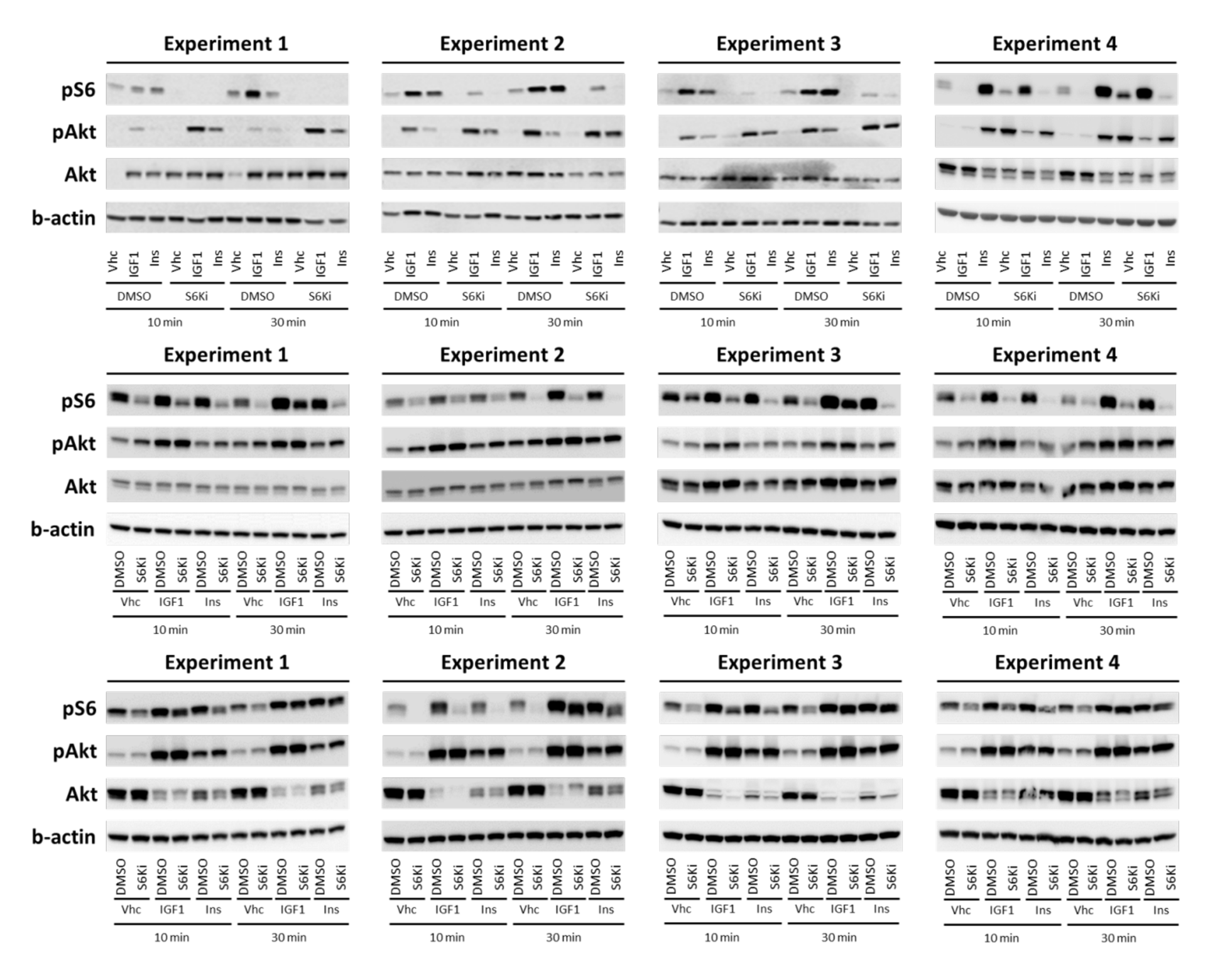

Supplement: S9 Fig — The RPS6K is inhibited in MCF7 (top row), T47D (middle row), and ZR75-1 (bottom row) cell lines. The pS6 levels are used as the proxy for S6K inhibition efficiency in un-stimulated cells. The total Akt and b-actin levels is used to normalize pAkt and pS6 levels, respectively. The perturbagen, ligand, and time point for each sample are listed below the blot images. (TIF) [file pcbi.1009125.s009.tif]

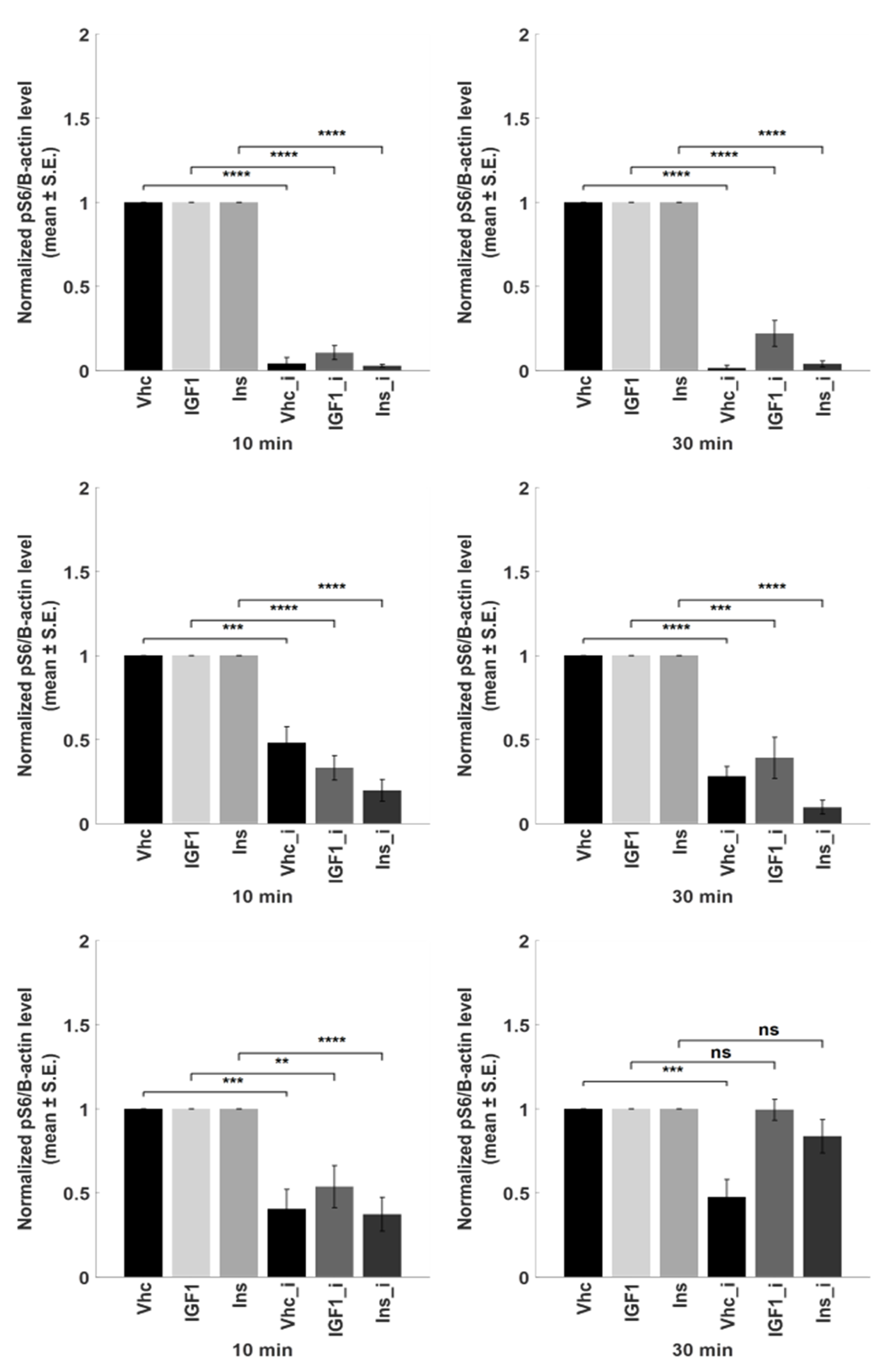

Supplement: S10 Fig — The RPS6K is inhibited in three cell lines of MCF7 (top row), T47D (middle row), and ZR75-1 (bottom row). The values are reported as normalized to the corresponding no-inhibition control. S6 phosphorylation quantification at 10 and 30 min show inhibition efficiency The results are compared using unpaired, one-tailed two-sample t-test, and P<0.05 (*), P<0.01 (**), P<0.005 (***), nonsignificant (ns). Results shown are mean ± s.e.m. of four independent replicates. (TIF) [file pcbi.1009125.s010.tif]

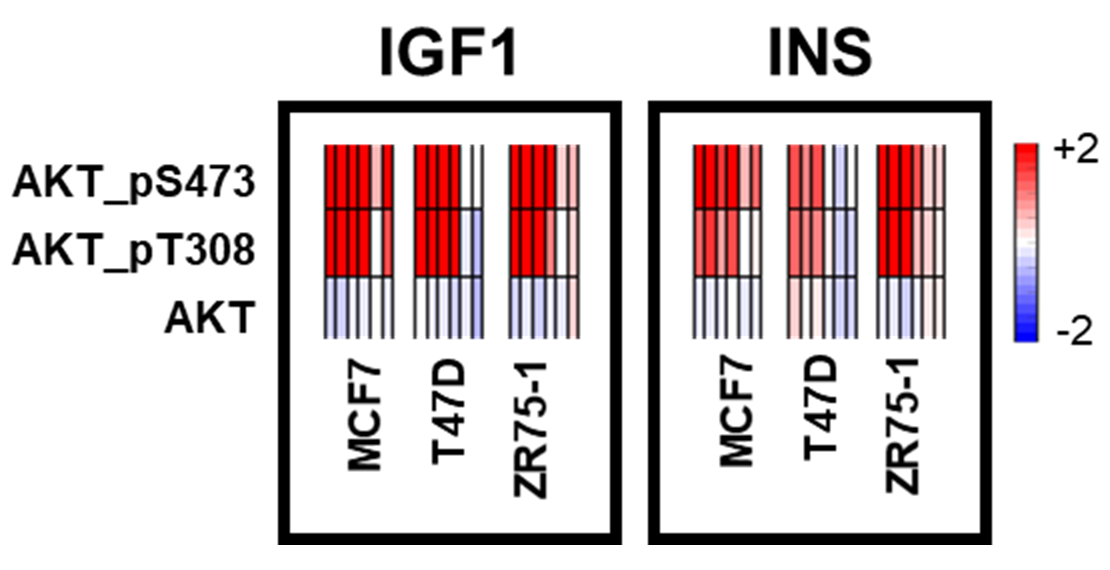

Supplement: S11 Fig — Time-course (phospho)-protein expression profiles. The change in the (phospho)-protein levels upon IGF1 or insulin stimulation, represents the difference in log2 expression levels (stimulated vs serum-starved). Six columns are shown for each cell line, each column representing one time point (5, 10, 30 min, 6, 24, 48 h). Red: up-regulation, blue: down-regulation. Adapted from [25]. (TIF) [file pcbi.1009125.s011.tif]
