# Supplementary material for: Inhibition of RPS6K reveals context-dependent Akt activity in luminal breast cancer cells
Source: PLoS Comput Biol. 2021 Jun 30;17(6):e1009125. doi: 10.1371/journal.pcbi.1009125 (PMC8277016; doi:10.1371/journal.pcbi.1009125)
Supplement: S4 Table — (DOCX) [file pcbi.1009125.s015.docx]

**S4 Table.** PTEMPEST parameter estimation configurations

| **Parameter settings name** | **Description** | **Value** |
| --- | --- | --- |
| nchains | number of chains | 4 |
| nswaps | number of chain swaps (=number of saved samples) | 50000 |
| nsteps | number of steps between chain swaps | 25 |
| adapt_last | last adaption step | 2900 |
| energy_init_max | maximum allowed energy for initialization | 1500 |
| beta_init | beta initialization parameter | 0.5 |
| relstep_init | relstep initialization parameter | 0.01 |
