# Supplementary material for: Inhibition of RPS6K reveals context-dependent Akt activity in luminal breast cancer cells
Source: PLoS Comput Biol. 2021 Jun 30;17(6):e1009125. doi: 10.1371/journal.pcbi.1009125 (PMC8277016; doi:10.1371/journal.pcbi.1009125)
Supplement: S5 Table — (DOCX) [file pcbi.1009125.s016.docx]

**S5 Table.** Initial parameter values of the model

| IGF1R_0 | 25000 | kf1c | 4.99968 | kf13 | -6.40681 | kf112 | -2.88861 |
| --- | --- | --- | --- | --- | --- | --- | --- |
| INSR_0 | 25000 | kf1d | -6.41314 | kf14 | -2 | kf201 | 1.399404 |
| IRS_0 | 120000 | kf2 | 2.237316 | kf15 | -6.28172 | kf202 | 4.949971 |
| SOS_0 | 120000 | kf2b | -1.55046 | kf16 | -7.05801 | kf203 | -7.59225 |
| RAS_0 | 120000 | kf2c | 4.99968 | kf17 | -2.93586 | kf204 | 2.288491 |
| RAF_0 | 120000 | kf2d | -6.41314 | kf101 | -2.77918 | kf206 | -0.67347 |
| MEK_0 | 600000 | kf3 | -4.80963 | kf102 | -2.1035 | kf207 | -0.81225 |
| ERK_0 | 600000 | kf4 | -4.53019 | kf103 | -0.02609 | kf208 | 2.106079 |
| PI3K_0 | 120000 | kf5 | -1.44464 | kf104 | -0.34845 | kf301 | 4.6656 |
| PDK1_0 | 120000 | kf6 | -2.77833 | kf105 | -6.58613 | kf302 | -5.39972 |
| AKT_0 | 600000 | kf7 | -3.60524 | kf106 | 2.654115 | kf303 | -4.82365 |
| TSC2_0 | 80000 | kf8 | -3.15563 | kf107 | -1.98143 | kf304 | -7.5594 |
| mTORC1_0 | 80000 | kf9 | -7.05415 | kf108 | 0.762129 | kf401 | -4 |
| RPS6K_0 | 80000 | kf10 | 1 | kf109 | -7.12815 | kf402 | -4 |
| kf1 | 2.237316 | kf11 | -1 | kf110 | 0 | kf403 | -3.05933 |
| kf1b | -1.55046 | kf12 | -3.35773 | kf111 | 2.161502 | kf404 | -4.14698 |
