# Supplementary material for: Inhibition of RPS6K reveals context-dependent Akt activity in luminal breast cancer cells
Source: PLoS Comput Biol. 2021 Jun 30;17(6):e1009125. doi: 10.1371/journal.pcbi.1009125 (PMC8277016; doi:10.1371/journal.pcbi.1009125)
Supplement: S1 Text — (DOCX) [file pcbi.1009125.s017.docx]

## S1 Text – Model parameter estimation and sensitivity analysis

### Parameter estimation protocol

Our constructed model (Fig 1A) had 66 parameters (16 were the total protein counts). The parameters are estimated using parameter ranges from the literature [1–4]. The protocol for parameter estimation included a semi-automated and piece-wise fitting strategy, using Markov chain Monte Carlo sampling and the PTEMPEST package (github.com/RuleWorld/ptempest). The PTEMPEST algorithm searches the parameter space running multiple Markov chains in parallel at different temperatures for faster sampling and enabling escape from the local minima [5]. In this framework, the probability of accepting an unfavorable move depends on the chain temperature and the swaps among different chains help avoid getting stuck in the local minima. The high temperature chains scan the parameter space more globally, and lower temperature chains enables localized searches. The approach samples the Bayesian posterior distribution of each parameter, with uniform priors [6,7]. The estimation procedure outputs parameter ensembles for each chain.

In our work, we have run four chains in parallel and allowed for 50000 swaps between chains in each run. S4 Table summarizes the full list of the parameter settings for PTEMPEST configuration.

Starting from the parameter values (S5 Table), we ran (sampled) a total of 1,250,000 parameter sets (25 x 50000 swaps). For each PTEMPEST run, we manually defined the list of parameters to fit and the “least-error” parameter set from each run is used as the starting parameter set for the next run. An example of the estimation error for different batches of fitting is shown S1 Fig. The minimum fitting error parameter set from the whole process was defined as the “best-fit” (S1 Table) and was used for all subsequent analyses.

### Sensitivity analysis

Each parameter in the “*best-fit*” set is varied individually, first halved and then doubled. With every individual change, the model is run to 30min. The time-course data for IGF1 and insulin stimulation are compared to the unperturbed stimulation results. There are two different measures of comparisons. First is the comparison of responses in all four observables at 5min. The plots showing all four phospho-protein results are in S4 and S5 Figs.

The second measure of comparisons is the ratio of area under the curve (AUC) values for each of the four proteins. The ratios are calculated dividing the perturbed model results by the un-perturbed simulation results. The plots are given in S6 and S7 Figs. The two measures showed similar and expected results, where parameters of specific cascades affect the corresponding phospho-protein levels almost exclusively.

### References

1. Sarma U, Ghosh I. Different designs of kinase-phosphatase interactions and phosphatase sequestration shapes the robustness and signal flow in the MAPK cascade. BMC Syst Biol. 2012;6(1):82.

2. Schoeberl B, Eichler-Jonsson C, Gilles ED, Muller G. Computational modeling of the dynamics of the MAP kinase cascade activated by surface and internalized EGF receptors. Nat Biotechnol. 2002;20:370–5.

3. Kocieniewski P, Lipniacki T. MEK1 and MEK2 differentially control the duration and amplitude of the ERK cascade response. Phys Biol. 2013 Jun 4;10(3):035006.

4. Creamer MS, Stites EC, Aziz M, Cahill JA, Tan C, Berens ME, et al. Specification, annotation, visualization and simulation of a large rule-based model for ERBB receptor signaling. BMC Syst Biol. 2012;6(1):107.

5. Gupta S, Hainsworth L, Hogg J, Lee R, Faeder J. Evaluation of Parallel Tempering to Accelerate Bayesian Parameter Estimation in Systems Biology. In: 2018 26th Euromicro International Conference on Parallel, Distributed and Network-based Processing (PDP). 2018. p. 690–7.

6. Swigon D. Ensemble Modeling of Biological Systems. In: Antoniouk A V., Melnik RVN, editors. Mathematics and Life Sciences. Walter de Gruyter; 2012. p. 19–42.

7. Brown KS, Sethna JP. Statistical mechanical approaches to models with many poorly known parameters. Phys Rev E. 2003 Aug 12;68(2):21904.
