# Supplementary material for: Inhibition of RPS6K reveals context-dependent Akt activity in luminal breast cancer cells
Source: PLoS Comput Biol. 2021 Jun 30;17(6):e1009125. doi: 10.1371/journal.pcbi.1009125 (PMC8277016; doi:10.1371/journal.pcbi.1009125)
Supplement: S3 Text — (PDF) [file pcbi.1009125.s019.pdf]

1 Model Summary

The model has 38 species and 50 reactions.

2 Species

$$
\begin{aligned}
x_1 &= IGF1 \\
x_2 &= Insulin \\
x_3 &= IGF1R \\
x_4 &= InsR \\
x_5 &= IRS \\
x_6 &= SOS \\
x_7 &= Ras \\
x_8 &= Raf \\
x_9 &= MEK \\
x_{10} &= PI3K \\
x_{11} &= PDK1 \\
x_{12} &= TSC2 \\
x_{13} &= mTOR \\
x_{14} &= Akt \\
x_{15} &= RPS6K \\
x_{16} &= ERK \\
x_{17} &= IGF1-IGF1R \\
x_{18} &= Ins-InsR \\
x_{19} &= IGF1-pIGF1R \\
x_{20} &= Ins-pInsR \\
x_{21} &= pIRS \\
x_{22} &= aSOS \\
x_{23} &= IGF1-pIGF1Rint \\
x_{24} &= Ins-pInsRint \\
x_{25} &= aRas \\
x_{26} &= aPI3K \\
x_{27} &= pRaf \\
x_{28} &= aPDK1 \\
x_{29} &= pMEK \\
x_{30} &= pAkt \\
x_{31} &= pERK
\end{aligned}
$$

$$
\begin{aligned}
x_{32} &= pTSC2 \\
x_{33} &= iRaf \\
x_{34} &= iIRS \\
x_{35} &= mTORact \\
x_{36} &= iSOS \\
x_{37} &= iMEK \\
x_{38} &= pRPS6K
\end{aligned}
$$

3 Differential Equations

$$
\begin{aligned}
\dot{x_1} &= -kf_{1a}x_1x_3 + kf_{1b}x_{17} \\
\dot{x_2} &= -kf_{2a}x_2x_4 + kf_{2b}x_{18} \\
\dot{x_3} &= -kf_{1a}x_1x_3 + kf_{1b}x_{17} + kf_{402}x_{23} \\
\dot{x_4} &= -kf_{2a}x_2x_4 + kf_{2b}x_{18} + kf_{404}x_{24} \\
\dot{x_5} &= -kf_3x_{19}x_5 - kf_4x_{20}x_5 + kf_{101}x_{21} - kf_{208}x_{30}x_5 \\
&\quad -kf_{206}x_{31}x_5 + kf_{301}x_{34} - kf_{203}x_{38}x_5 \\
\dot{x_6} &= -kf_5x_{19}x_6 - kf_6x_{20}x_6 - kf_7x_{21}x_6 + kf_{102}x_{22} \\
&\quad -kf_{201}x_{31}x_6 + kf_{302}x_{36} \\
\dot{x_7} &= -kf_8x_{22}x_7 + kf_{103}x_{25} \\
\dot{x_8} &= -kf_9x_{25}x_8 + kf_{104}x_{27} - kf_{204}x_{30}x_8 + kf_{303}x_{33} \\
\dot{x_9} &= -kf_{10}x_{27}x_9 + kf_{105}x_{29} - kf_{202}x_{31}x_9 + kf_{304}x_{37} \\
\dot{x_{10}} &= -kf_{12}x_{21}x_{10} + kf_{106}x_{26} \\
\dot{x_{11}} &= -kf_{13}x_{26}x_{11} + kf_{107}x_{28} \\
\dot{x_{12}} &= -kf_{15}x_{30}x_{12} + kf_{108}x_{32} \\
\dot{x_{13}} &= -kf_{16}x_{32}x_{13} + kf_{109}x_{35} \\
\dot{x_{14}} &= -kf_{14}x_{28}x_{14} + kf_{110}x_{30} + kf_{207}x_{31}x_{30} \\
\dot{x_{15}} &= -kf_{17}x_{35}x_{15} + kf_{111}x_{38} \\
\dot{x_{16}} &= -kf_{11}x_{29}x_{16} + kf_{112}x_{31} \\
\dot{x_{17}} &= +kf_{1a}x_1x_3 - kf_{1b}x_{17} - kf_{1c}x_{17} + kf_{1d}x_{19} \\
\dot{x_{18}} &= +kf_{2a}x_2x_4 - kf_{2b}x_{18} - kf_{2c}x_{18} + kf_{2d}x_{20} \\
\dot{x_{19}} &= +kf_{1c}x_{17} - kf_{1d}x_{19} - kf_{401}x_{19} \\
\dot{x_{20}} &= +kf_{2c}x_{18} - kf_{2d}x_{20} - kf_{403}x_{20} \\
\dot{x_{21}} &= +kf_3x_{19}x_5 + kf_4x_{20}x_5 - kf_{101}x_{21} \\
\dot{x_{22}} &= +kf_5x_{19}x_6 + kf_6x_{20}x_6 + kf_7x_{21}x_6 - kf_{102}x_{22} \\
\dot{x_{23}} &= +kf_{401}x_{19} - kf_{402}x_{23} \\
\dot{x_{24}} &= +kf_{403}x_{20} - kf_{404}x_{24}
\end{aligned}
$$

$$
\begin{aligned}
\dot{x_{25}} &= +kf_8 x_{22} x_7 - kf_{103} x_{25} \\
\dot{x_{26}} &= +kf_{12} x_{21} x_{10} - kf_{106} x_{26} \\
\dot{x_{27}} &= +kf_9 x_{25} x_8 - kf_{104} x_{27} \\
\dot{x_{28}} &= +kf_{13} x_{26} x_{11} - kf_{107} x_{28} \\
\dot{x_{29}} &= +kf_{10} x_{27} x_9 - kf_{105} x_{29} \\
\dot{x_{30}} &= +kf_{14} x_{28} x_{14} - kf_{110} x_{30} - kf_{207} x_{31} x_{30} \\
\dot{x_{31}} &= +kf_{11} x_{29} x_{16} - kf_{112} x_{31} \\
\dot{x_{32}} &= +kf_{15} x_{30} x_{12} - kf_{108} x_{32} \\
\dot{x_{33}} &= +kf_{204} x_{30} x_8 - kf_{303} x_{33} \\
\dot{x_{34}} &= +kf_{208} x_{30} x_5 + kf_{206} x_{31} x_5 - kf_{301} x_{34} + kf_{203} x_{38} x_5 \\
\dot{x_{35}} &= +kf_{16} x_{32} x_{13} - kf_{109} x_{35} \\
\dot{x_{36}} &= +kf_{201} x_{31} x_6 - kf_{302} x_{36} \\
\dot{x_{37}} &= +kf_{202} x_{31} x_9 - kf_{304} x_{37} \\
\dot{x_{38}} &= +kf_{17} x_{35} x_{15} - kf_{111} x_{38}
\end{aligned}
$$
