# Supplementary material for: Inhibition of RPS6K reveals context-dependent Akt activity in luminal breast cancer cells
Source: PLoS Comput Biol. 2021 Jun 30;17(6):e1009125. doi: 10.1371/journal.pcbi.1009125 (PMC8277016; doi:10.1371/journal.pcbi.1009125)
Supplement: Attachment — Submitted filename: MMpaper_rebuttal_PLOScb_v2.docx [file pcbi.1009125.s020.docx]

**Reviewer #1:**

For much of the paper the exact modelling methodology used is unclear. The paper would be improved if there was a clear description of the BioNetGen/RuleBender/ODE based modelling approach used and an overview of model training against proteomics data upfront. The input, output, training and semantics of the model are not made precise as early as they could be. Some of this detail is already in the discussion and methods sections.

We appreciate this suggestion and agree with the Reviewer that an upfront layout of our modeling procedure will clarify our work to readers. We have added descriptive text in the Introduction (lines 82-86) to present our methods.

The discussion would benefit from comments on the implications of these results for breast cancer therapy.

We have included new text in the Discussion (lines 260-268) to address the broader impacts of our work in the realm of breast cancer therapy.

**Reviewer #2:** 

The authors base their study on a number of previous studies and modelling efforts, and for this particular effort concentrate their mechanistic model on a few pathways that are downstream of both the IGF1R and the InsR. The main signalling axes encompasses the Akt-mTor axis, as well as the Ras-Raf-Mapk pathways, including several feedback-loops between them. This might seem a reductionist approach, but to me it appears an appropriate approach for a targeted question. This assertion is largely vindicated in the correct prediction of cellular responses to IGF1 stimulation in Fig 2B. However, it is apparent that the model consistently over-estimates the effect of small amounts of IGF1 on the downstream pathways. Biological systems frequently operate with thresholds, and possibly the model could be improved, by adding a threshold before which the downstream pathways are not triggered. If this is not feasible, this discrepancy should at least be addressed and explained.

We have added text (lines 167-174) to address possible reasons for the fitting mismatch at lower IGF1 doses. Since the data here were a test set (i.e., they were not included in model training), we believe the Reviewer would agree that such deviations are expected and acceptable as no further data-fitting was employed. The RPPA data on which the model was trained were generated using an IGF1 concentration of 10nM. The agreement between model and experiment at 10nM of IGF1 demonstrates that the model successfully re-captures new independent data with a similar context as the training data. We thus believe no further modifications to the model were necessary at this time, and we edited the text to explain this.

One of the central tenets of the authors is that the two stimulatory pathways differ from each other insofar as S6K inhibition leads to a further increase in AKT phosphorylation downstream of Ins stimulation 30 minutes post exposure, while the same is not true for IGF1 stimulation. They perform a number of validation experiment in a number of cell lines (Fig. 4), which seemingly confirm this idea. The main problem I see with this set of experiments, is that in all instances the initial AKT phosphorylation following IGF1 stimulation is significantly higher than following Ins stimulation. This makes the effects hard to compare, as pAKT might already have reached maximum phosphorylation levels after 10 minutes of IGF1 stimulation combined with S6K inhibition, with no chance of rising even higher within the following 20 minutes of incubation. Alternatively, completely independent feedback mechanisms might be at play, as biological systems tend to be self-regulatory. In fact, in the case of MCF7 cells, where the difference between the initial levels of pAKT elicited by IGF1 or Ins is not quite as big as in the other cell lines, a clear trend towards a further increase of pAKT after 30 minutes of IGF1 stimulation with S6K inhibition can be seen.

We agree that such a mechanism of reaching maximal phosphorylation upon IGF1 treatment is possible and can cloud our observations. This same concern was one of the major reasons why we performed validation experiments in two additional cell lines (T47D and ZR75-1) together with MCF7. As shown in the Figure below (adapted from our earlier work where profiled multiple cell lines [25]), the phosphorylation profiles in ZR75-1 cells with 10 nM IGF1 and Ins are very close each other, as the Reviewer would like to see. The inhibition of p70S6K in these cells also conveyed a more significant pAKT increase in insulin-stimulated cells. We thus believe that our current results already answer the Reviewer’s point, and these experimental efforts confirmed the model predictions. We added a discussion text (lines 260-268) and the below figure (Fig. S11) to articulate this concern in the manuscript.

Time-course (phospho)-protein expression profiles. The change in the (phospho)-protein levels upon IGF1 or insulin stimulation, represents the difference in log2 expression levels (stimulated vs serum-starved). Six columns are shown for each cell line, each column representing one time point (5, 10, 30 min, 6, 24, 48 h). Red: up-regulation, blue: down-regulation. Adapted from [25].

Personally, I struggle to believe that significance has not been reached, when comparing control cells to inhibitor treated ones at 30 minutes (top right panel).

We also believe that is the case visually, but the p-values for IGF1 fall short of significance. Please also note that the error bars are s.e.m not std. Here are the corresponding p-values from unpaired, one-tailed two-sample t-test:

p-values = [0.1337-IGF, 0.0013-Ins] for 10min & [0.0958-IGF, 0.0028-Ins] for 30min

I thus propose to repeat at the experiments in at least one cell line, while titrating IGF1 concertation such that it gives an equivalent AKT phosphorylation as Ins stimulation.

As we explained above, we believe showing our model predictions hold true for three different cell lines with different “normal” pAKT response profiles was satisfactory enough, and no further experiments are necessary for this work at this time.
